# City Centrality, Migrants and Green Inovation Efficiency: Evidence from 106 Cities in the Yangtze River Economic Belt of China

**DOI:** 10.3390/ijerph17020652

**Published:** 2020-01-20

**Authors:** Haisen Wang, Gangqiang Yang, Jiaying Qin

**Affiliations:** 1 Institute for the Development of Central China, Wuhan University, Wuhan 430072, China; hswang@whu.edu.cn (H.W.); jyqin123@whu.edu.cn (J.Q.); 2 Development Research Center of the Yangtze River Economic Belt, Wuhan University, Wuhan 430072, China

**Keywords:** city centrality, green innovation efficiency, gravity model, Yangtze River Economic Belt

## Abstract

Based on the panel data of 106 cities in the Yangtze River Economic Belt of China from 2007 to 2016, this paper explores the impact of city centrality on the green innovation efficiency and proves the mediation effect of migrants by using spatial econometric model. The results show that there are more and more innovation contacts between cities, and the innovation network is becoming more and more dense. The core cities of the downstream innovation network are mainly Yangzhou, Zhenjiang, Wuxi, Changzhou, Suzhou and Hangzhou; the core cities in the midstream are mainly Wuhan, Changsha and Yichun; the core cities in the upstream are Chengdu and Bazhong. There is an inverted U-shaped relationship between city centrality and green innovation efficiency. In addition, the influence curve of city centrality on the green innovation efficiency of surrounding cities is also inverted U-shaped. Cities with high city centrality attract a large number of migrants that come from cities with lower centrality to improve the green innovation efficiency, but the green innovation efficiency of cities with low city centrality will decline due to lack of talents.

## 1. Introduction

Since the reform and opening up, the Yangtze River Economic Belt has became one of the regions with the strongest comprehensive strength in China. However, the development of the Yangtze River Economic Belt faces many difficulties and problems that need to be solved urgently, which are mainly the severe situation of the ecological environment, the arduous task of industrial transformation and upgrading, and the imperfect mechanism of regional innovation cooperation. Therefore, effectively improving the green innovation efficiency in the Yangtze River Economic Belt and giving full play to its role as a green economic support belt will help to improve these problems.

China is in a stage of high-quality development, innovation and green development play important role in the national economic strategy. From the perspective of traditional innovation concept, the scholars who first introduced environmental factors into the field of innovation proposed four kinds of innovations in turn: ecological innovation, environmental innovation, sustainable innovation and green innovation. The discussion of ecological innovation originated with Fussler and James [1], who defined ecological innovation as “new products and processes that provide business value to customers but significantly reduce environmental impact.” In the early 1920s, environmental innovation research was still at a nascent stage, and few researchers worldwide who studied innovation addressed environmental problems until Oltra and Jean defined environmental innovation as “New or improved processes, practices, systems and products that benefit the environment [2].” Sustainable innovation increasingly implies long-term and far-reaching changes in technology, infrastructure, lifestyles and institutions. With the rapid growth of the world’s population, living space has been dramatically reduced, and traditional production, manufacturing and business models largely cannot keep up with the pace of economic development. Natural resources are increasingly scarce, and environmental damage is becoming increasingly serious [3]. To reduce environmental pollution and energy consumption, countries are paying growing attention to green technology innovation to achieve the maximum economic and ecological benefits with the lowest levels of consumption and pollution [4]. Chen et al. define green innovation as “hardware or software innovation related to green products or processes, which include technological innovation related to energy conservation, pollution prevention, waste recycling and green product design [5].” Innovation is the fundamental driving force for promoting economic and social development, and green technology innovation is the key driving force for achieving low-carbon economic development and improving the efficiency of natural resources [6]. China has always placed innovation in an important position in the overall development of the country. Relevant data show that the number of R&D personnel in China increased from 304,000 in 2008 to 462,000 in 2017. Internal R&D expenditures over the same period increased from 68.79 billion yuan to 243.57 billion yuan, with an average annual growth rate of 15.08%. Bai [7], however, notes that increasing R&D is a necessary condition for building an innovative country; the focus should be not only on the amount of R&D investment but also on the efficiency of innovation, it is equally salient for developing countries with low levels of science and technology. Green innovation efficiency is a comprehensive efficiency index that considers innovation performance, resource and environmental constraints [8]. Previous studies on green innovation mainly focus on measuring the level of green innovation [9], dividing the types of green innovation [10], analyzing the influencing factors of green innovation [11]. These scholars only explore the influence of regional internal factors on green innovation efficiency without considering the spillover effect of innovation [12,13]. Innovation contacts between regions have complex and network-like structure, and the mobility of R&D elements and the strong connection between researchers make the connectivity of the technological innovation network come true, network research is also applicable to innovation systems [14].

Therefore, this article explores green innovation efficiency from the perspective of social network theory. With the increasing complexity of innovation activities, the increasing number of R&D processes is often a collective act, as it reduces the risks and complexities involved in developing new products and processes by spreading innovation to dispersed partners [15]. The evolution of innovation spatial patterns to network has led to the transformation of urban innovation function and innovation mode. Previous scholars have discussed the urban innovation network from the perspective of network structure characteristics and spatial attributes [16,17,18]. However, these studies only describe the characteristics of innovation networks and the mechanism of dynamic evolution, and the research on how network structure elements promote city innovation is still lacking. This paper quotes the research of enterprise innovation networks, which mainly shows that the innovation of individuals or higher-level collectives (teams, organizations, or countries) is influenced by their social network. These social relationships and networks can promote or constrain them to acquire, transfer, absorb, evaluate, and apply knowledge and information [19]. For example, Chen et al. [20] analyzed how an innovation network structure enhances or constrains innovation activities by affecting the frequency of interactions between members and knowledge within the network. Olanrewaju et al. use centrality to represent the number of other enterprises that enterprises associate with through informal social relationships—the higher the enterprise’s centrality, the stronger its ability to obtain network resources, which can allow the enterprise to obtain and utilize the knowledge spillover related to innovation as much as possible [21]. Rydehell et al. also claimed that the position of the company in the innovation network is a key factor affecting the innovation performance of the company [22]. The above studies have shown that the member in the center of the network can make better use of information resources to extract more valuable technology and knowledge, so as to realize the progress and innovation of their own technology. However, this conclusion seems to be more commonly applied to corporate cases, and few scholars have used data of cities to explore this issue. City centrality is used to represent the status of a city in the city innovation network, so we attempt to explore whether there is a similar relationship between city’s green innovation efficiency and city centrality, and what is the mechanism of this correlation. This is the research question of this article. Therefore, this paper proposes the first hypothesis:

**Hypothesis** **1.** *City centrality affects green innovation efficiency.*

Some researchers believe that heterogeneity in innovation performance comes from the abilities to access and create the resources [23], when using external knowledge resources to innovate, it’s easy for enterprises to produce innovation without the guidance of partners if innovative resources can be fully absorbed and utilized [24]. Dong found that the acquisition of innovative resources depends on knowledge network centrality [25]. Therefore, the centrality increases the chances of acquiring innovative resources [26], the innovation network plays an important role for network members to acquire innovation resources through inter-organizational relationships, and the positions of members in the network structure directly affect their ability to acquire resource. The same is true for city innovation. With the deepening of social aging and the continuous decline of the number of labor resources, the problem of structural shortages in the labor market is becoming increasingly serious [27]. In this context, how to attract talents and how to “rob” high-quality labor become the key to the future development of the city, various cities are also competing to introduce preferential policies to attract talents, while policies issued by some developed cities are more attractive to talents, and these cities tend to have higher innovation level and higher city centrality, so cities that are at the center of the innovation network seem to be more likely to acquire higher skilled labors or more capital investment, so this paper proposes the second hypothesis:

**Hypothesis** **2.** *Cities with high city centrality attract a large number of migrants that come from cities with low city centrality to improve the green innovation efficiency*.

Based on two hypotheses, this paper first uses the spatial econometric model to explore the impact of city centrality on the green innovation efficiency from the perspective of city network, heterogeneity test is used to analyze the different effects of city centrality. Second, the mediation effect model is used to prove the number of migrants is a mediator variable in the effect of the city centrality promoting the green innovation efficiency. Last, the above conclusions still hold after considering the robustness test. The paper proceeds in the following way: the second section introduces materials and methods, the third section describes the results, the fourth section discusses the results, and the last section provides the conclusions and policy recommendations.

## 2. Materials and Methods

### 2.1. Variables

#### 2.1.1. Dependent Variable

This paper calculates the green innovation efficiency by using MAX-DEA software. In 1978, Charnes et al. first proposed a nonparametric technical efficiency analysis method based on a relative comparison between the evaluated decision-making units (DMUs)—DEA [28]. The SBM model proposed by Tone solves the problem posed by the fact that radial models do not contain slack variables in their measurement of inefficiency [29]. SBM model cannot distinguish DMUs with an efficiency of 1, but a super-efficiency model can rank DMUs with efficiency at the frontier. Therefore, the super-SBM model is further defined by the fact that it combines the advantages of the super-efficiency model and the SBM model. The model is as follows:

(1) minρSE=1m∑i=1mxi¯xik1s∑r=1syr¯yrks.t. xi¯≥∑j=1,j≠knxijλj; yr¯≤∑j=1,j≠knyrjλj; xi¯≥xik; yr¯≤yrk; λ,s−,s+,y¯≥0;  i=1,2,⋯,m; r=1,2,⋯,q; j=1,2,⋯,n(j≠k)

Here, n is the number of DMUs, where each DMU has m inputs and q outputs, which are denoted as $x_{i}\left(i=1,2,\ldots ,m\right)$ and $y_{r}\left(r=1,2,\ldots ,q\right)$, respectively. λ represents the linear combination coefficient of the DMUs, and the specific indicators are selected as follows.

Input indicators: Independent innovation or the purchase of extraterritorial technology requires R&D personnel and financial resources as inputs, which need the support of local governments, funding and staffing must be guided by the number of topics [30]. Based on available data, this paper uses the fixed asset investment ($X_{1}$), the number of employees in the second and third industries ($X_{2}$), science and technology expenditure ($X_{3}$) and electricity consumption ($X_{4}$) as input indicators.

Output indicators: The output of green innovation can be measured in two ways: technological output and green output. Technological output can be further divided into two processes: achievements of technological creation and achievements of technological transformation, The number of patent authorizations ($Y_{1}$) has been shown to better represent the quality of innovation output [31]. Achievements of technological transformation are premised on harvesting economic benefits; the ultimate goal of technological innovation is to pursue the application of academic achievements and to obtain economic benefits by selling new products [32]. Therefore, this paper chooses gross domestic product ($Y_{2}$) to represent achievements of technological transformation. In addition, the environmental pollution in the process of innovation activities is mainly caused by the discharge of industrial wastes [33]. Therefore, the emission of three pollutants ($y_{1}$ represents Industrial smoke and dust emissions, $y_{2}$ represents Industrial wastewater discharge, $y_{3}$ represents Industrial sulfur dioxide emissions) in industrial production is regarded as undesirable output. 

#### 2.1.2. Explanatory Variables

The core explanatory variable of this paper is the city centrality, the greater the centrality, the stronger the ability of the city to control other cities and the more innovation contacts between the city and other cities. This paper uses degree centrality to represent city centrality, degree centrality characterizes the ability of a node to contact other nodes in the network, the larger the value, the more prominent the centrality of the node in the network and the stronger the communication ability, the calculation formula is as follows:

(2) $$DC_{i}=\sum_{j=1}^{N}S_{ij}$$

where DC is the city centrality, which represents the sum of the number of innovation contacts between city *i* and all other cities, N is the number of cities in the network, $S_{ij}$ represents the number of innovation contacts between city *i* and city *j*, it’s measured by a modified gravity model. The calculation formula of the traditional gravity model is as follows:

(3) $$F_{ij}=\frac{G\cdot M_{i}\cdot M_{j}}{D_{ij}^{2}}$$

Here, $F_{ij}$ is the attraction of the region *i* to the region *j*, for example, G is the coefficient of attraction between regions, which is generally 1; $M_{i}$ and $M_{j}$ are some factors; $D_{ij}$ is the spatial straight-line distance between region *i* and region *j*. We modified the traditional gravity model, the innovation contacts are directly proportional to the innovation level of city *i* and city *j*, that is, if the two cities have higher levels of innovation, there will be more innovation interaction and knowledge exchange between them. The innovation contacts and the distance between the two cities will be inversely, because higher transportation costs and time costs limit the innovation interaction between the two cities. If the difference in economic development between the two cities is relatively large, the frequency of innovation interaction between the two cities is relatively low. The innovation contacts between cities are set as the function of the spatial distance and the number of patent authorizations, and the innovation contacts between cities decrease with distance and increase with the number of patent authorizations, and developed cities tend to have more innovation links with other cities, so the model is as follows:

(4) $$S_{ij}=K\cdot \frac{PA_{i}\cdot PA_{j}}{D_{ij}^{2}},K=\frac{GDP_{i}}{GDP_{i}+GDP_{j}}$$

Here, $PA_{i}$ and $PA_{j}$ denote the number of patent authorizations of city *i* and city *j*, respectively; $GDP_{i}$ denotes the gross domestic product; $D_{ij}$ denotes the spatial straight-line distance between city *i* and city *j*, which is measured by ArcGIS software.

#### 2.1.3. Control Variables

Based on reviewing the relevant literature [34,35], the following four factors are selected as control variables. Companies have the incentive to change production methods and use green technologies because of the increase of production cost caused by environmental regulation, which often motivate enterprises innovation to improve the innovation of cities, this paper chooses the comprehensive utilization rate of industrial solid waste (ER) to indicate the intensity of environmental regulation. Due to the differences in the knowledge base and innovation process of different industries, regions with higher industrial structure have higher innovation ability [36], so the proportion of the added value of the tertiary industry in the regional GDP is used to reflect the industrial structural characteristics of a region (IS); in China, the local economic development level and the local financial input directly affects the government’s support for science and technology activities, which are inseparable from the operation of regional innovation systems [37], thus, per capita gdp (PCGDP) and local fiscal expenditureare (GOV) are used to represent the economic development level and government support. We transform each variable logarithmically to eliminate the effects of heteroskedasticity.

#### 2.1.4. Mediator Variable

Wage and geographical distance significantly affect labors’ migration. If city *j* has higher wages than city *i*, then the labor of city *i* will flow into city *j* under the drive of “utility maximization”. If the distance between city *i* and city j is relatively long, then the labor does not necessarily choose to flow due to transportation cost and time cost. Therefore, this paper uses the modified gravity model to define the number of migrants:

(5) $$PFL_{ij}=\ln L_{j}\cdot \ln WAGE_{i}\cdot D_{ij}^{-2}$$

In the above formula, $PFL_{ij}$ reprensents the number of migrants flowing from city *j* to city *i*, $L_{j}$ represents the number of employees in the secondary and tertiary industriesand, and $WAGE_{i}$ represents the average wage of employees in city *i*. Therefore, the total amount of migrants of city *i* is as follows:

(6) $$PFL_{i}=\sum_{j=1}^{n}PFL_{ij}$$

### 2.2. Model

#### 2.2.1. Benchmark Model

Before applying the spatial econometric model, it is necessary to do a spatial correlation test to ensure the validity of the model. Geary’s C proposed by Geary is a more suitable test method, which is used to determine whether economic activities have global spatial correlation. The calculation formula is put in the Appendix A. 

Compared with the traditional econometric model, the spatial econometric model takes into account the spatial correlation commonly found in economics, that is, the sample observations in one area depend on the observations in other areas, and spatial correlation is reflected in the lag term of dependent variable and error term in spatial econometric model. Considering that there may be a spatial correlation of innovation activities among regions, one region may be affected by the innovation activities in other adjacent or non-adjacent regions. Thus, traditional econometric models may ignore the spatial correlation of the green innovation efficiency. Accordingly, this paper chooses relevant data from 106 cities in the Yangtze River Economic Belt and uses spatial econometric analysis technology to explore the relationship between city centrality and green innovation efficiency. According to the judgment rules proposed by Anselin et al. [38], the model is set up and fitted by maximum likelihood (ML) estimation and the likelihood-ratio (LR) test. In theory, any factors related to innovation activities may have an impact on innovation efficiency. The spatial lag model (SLM) is generally used to explore the spatial spillover effect in one region, in general, a spatial lag model is applicable if the dependent variable of a spatial unit depends on the dependent variable of the previous period of its surrounding spatial units [39]. In other words, the city’s $GIE_{t}$ is affected by the surrounding cities’ $GIE_{t-1}$. In general, unobservable variables are ubiquitous, such as regional accessibility, reputation, and status. If the regression model includes unobservable spatial autocorrelation effect, then the spatial error model is applicable, which assumes that spatial spillover are caused by random impact and their spatial effects are mainly transmitted by error terms. If the dependent variable of city *i* also depends on the independent variables of other cities, the spatial durbin model (SDM) is applicable, which takes into account the transmission mechanism of the above two models, it incorporates the spatial correlation of independent variables and dependent variables, and the spatial spillover effect is characterized by spatial interaction terms. If there is no spatial autocorrelation in the green innovation efficiency among cities, then a traditional ordinary least squares (OLS) model is used to analyze it. If there is spatial autocorrelation, then the optimal spatial econometric model must be further screened through testing. According to the test results, SDM is more suitable for analyzing the problems in this article. The specific reasons will be explained in the Section 4. The SDM is as follows:

(7) $$GIE_{it}=\beta_{0}+\delta WGIE_{it}+\beta_{1}DC_{it}+\beta_{2}DC_{it}^{2}+\beta_{3}X_{it}+\theta_{1}WDC_{it}+\theta_{2}WDC_{it}^{2}+\theta_{3}WX_{it}+\varepsilon_{\mathrm{it}}$$

Here, δ is the spatial autoregressive coefficient, which is used to measure the impact of other cities’ $GIE_{t-1}$ on the local $GIE_{t}$. $GIE_{it}$ is the green innovation efficiency of city *i* in year *t*, DC is the city centrality, X represents the control variable, which is as follows: ER, IS, PCGDP and GOV. W is the spatial distance weight matrix (Each element in the matrix is equal to the linear distance between the city *i* and the city *j*). ε represents perturbation terms subject to independent and identical distribution.

#### 2.2.2. Mediation Effect Model

In order to further explore the mechanism of city centrality affecting the green innovation efficiency, this paper refers to the mediation effect test method proposed by Baron and Kenny [40], and the model is set as shown in Equations (7)–(9).

(8) $$PFL_{it}=\delta_{1}+\delta_{2}DC_{it}+\mu_{it}+\varepsilon_{it}$$

The next is as follow:

(9) $$\begin{array}{ll} GIE_{it}= & \alpha_{0}+\phi WGIE_{it}+\alpha_{1}DC_{it}+\alpha_{2}DC_{it}^{2}+\alpha_{3}X_{it}+\alpha_{4}PFL_{it}+\varphi_{1}WDC_{it}\\ & +\varphi_{2}WDC_{it}^{2}+\varphi_{3}WX_{it}+\varphi_{4}WPFL_{it}+\varepsilon_{\mathrm{it}} \end{array}$$

Here, $PFL_{it}$ represents total migrants of city *i*, the coefficient ($\beta_{1}$) of Equation (7) is the total effect of city centrality on green innovation efficiency; the coefficient ($\delta_{2}$) of Equation (8) is the effect of city centrality on mediator variable (PFL); the coefficient ($\alpha_{4}$) of Equation (9) is the effect of mediator variable (PFL) on green innovation efficiency after controlling the influence of city centrality; the coefficient ($\alpha_{1}$) is the direct effect of city centality on the green innovation efficiency after controlling the effect of the mediator variable (PFL). Equation (9) can be tested under the condition that the regression coefficient estimates of DC in Equations (7) and (8) are significant. In Equation (9), if the regression coefficient estimate of DC is not significant, but the regression coefficient estimate of PFL is significant, indicating that PFL has a complete mediation effect; if both are significant, but the regression coefficient estimate of DC is smaller than the regression coefficient estimate of PFL, then PFL has a partially mediation effect.

### 2.3. Data Sources

The Yangtze River Economic Belt covers 11 provinces and cities including Shanghai, Jiangsu, Zhejiang, Anhui, Jiangxi, Hubei, Hunan, Chongqing, Sichuan, Yunnan, Guizhou, etc., with an area of about 2.0523 million square kilometers, which accounts for 21.4% of the country’s population. The Yangtze River Economic Belt spans three major regions of China’s eastern, central, and western regions, and has unique advantages and great development potential. Based on the availability of data, the relevant data of 106 cities in the Yangtze River Economic Belt from 2007 to 2016 were selected for the analysis. All data are drawn from the EPS database (http://olap.epsnet.com.cn/) and Chinese Research Data Services Platform (CNRDS, https://www.cnrds.com/Home/Login). Descriptive statistics for the data are shown in Table 1.

## 3. Results

**Figure 1:**
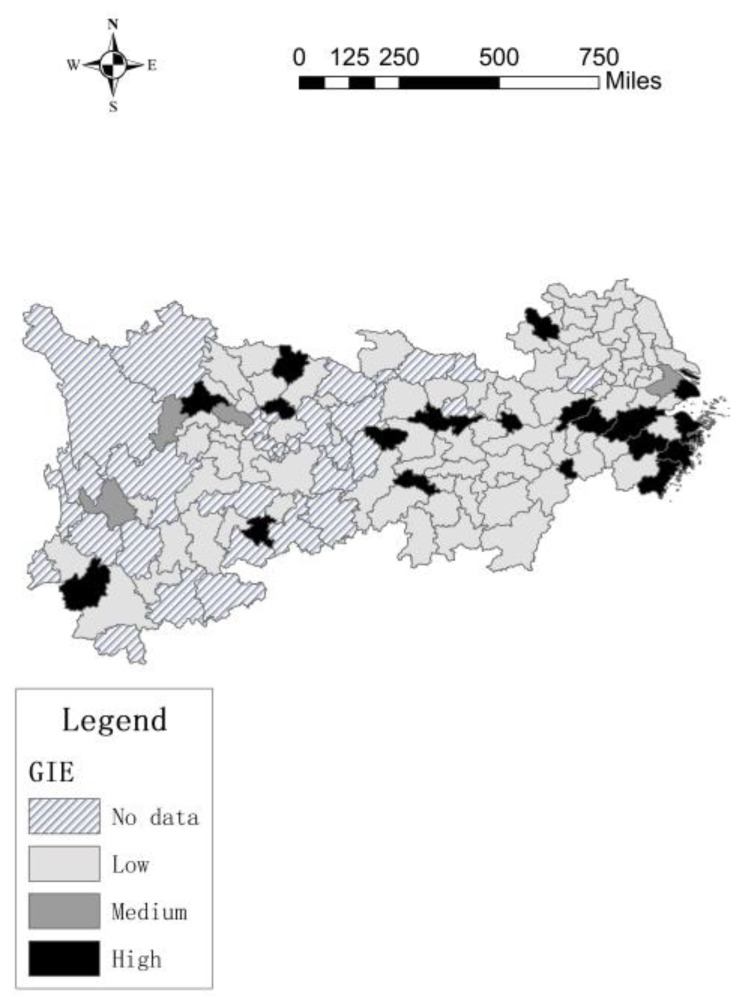
Green innovation efficiency in 2007.

**Figure 2:**
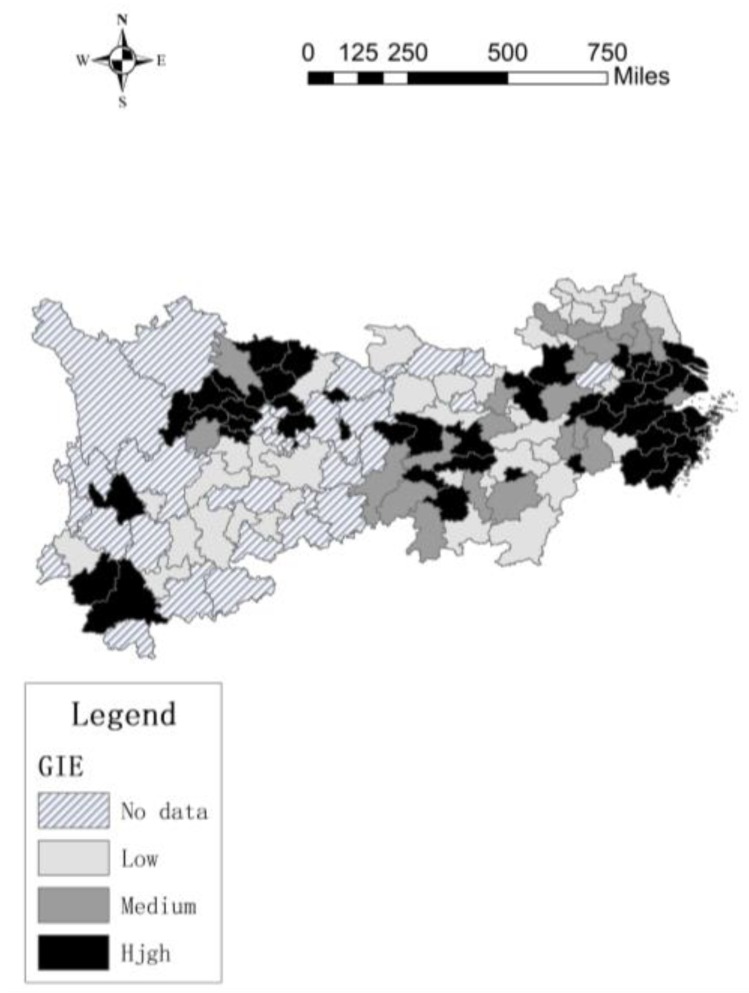
Green innovation efficiency in 2016.

MaxDEA software is used to build a super-SBM model with undesirable output to measure the green innovation efficiency of 106 cities. Additionally, this paper use different colors to show differing efficiency values across cities by ArcGIS, as shown in Figure 1 and Figure 2.

Figure 1 and Figure 2 show that, the green innovation efficiency of 106 cities in the Yangtze River Economic Belt was generally low in 2007. The green innovation efficiency of downstream cities was relatively high, such as Shanghai, Jiangsu and Wuxi, the differences between other cities were not obvious. However, in 2016, the overall green innovation efficiency of the Yangtze River Economic Belt has increased significantly, especially in the downstream cities. At th same time, the green innovation efficiency in the midstream and upstream cities has increased, but the gap between the upper, middle and lower reaches has also increased, this may be due to the agglomeration effect, the cities with high green innovation efficiency drive innovation in surrounding cities.

**Figure 3:**
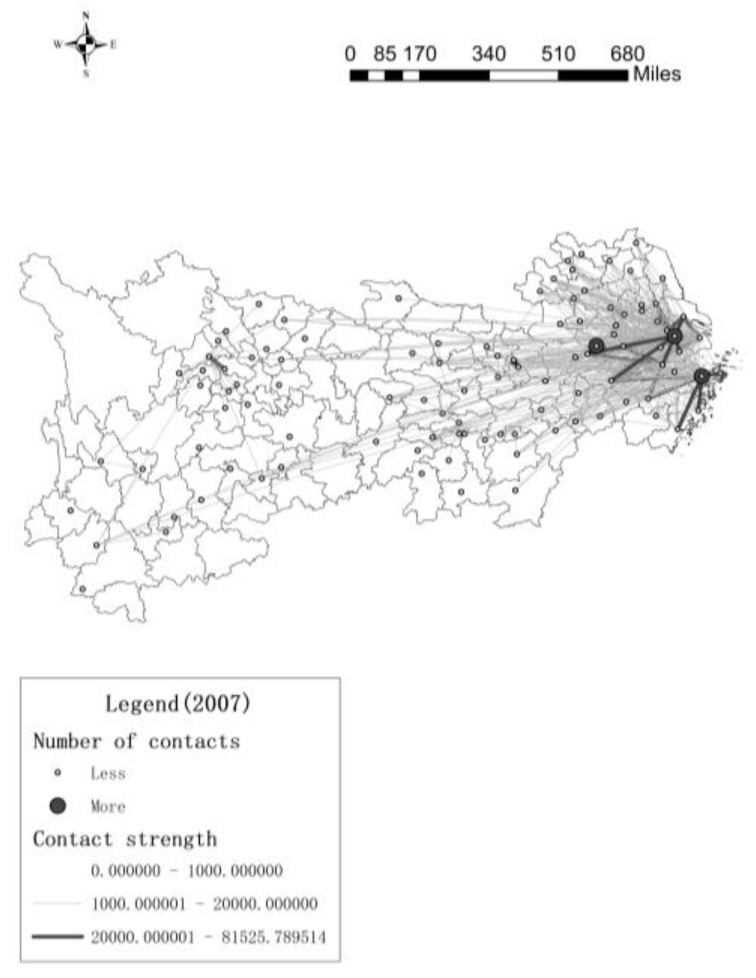
Number of urban innovation contacts in 2007.

**Figure 4:**
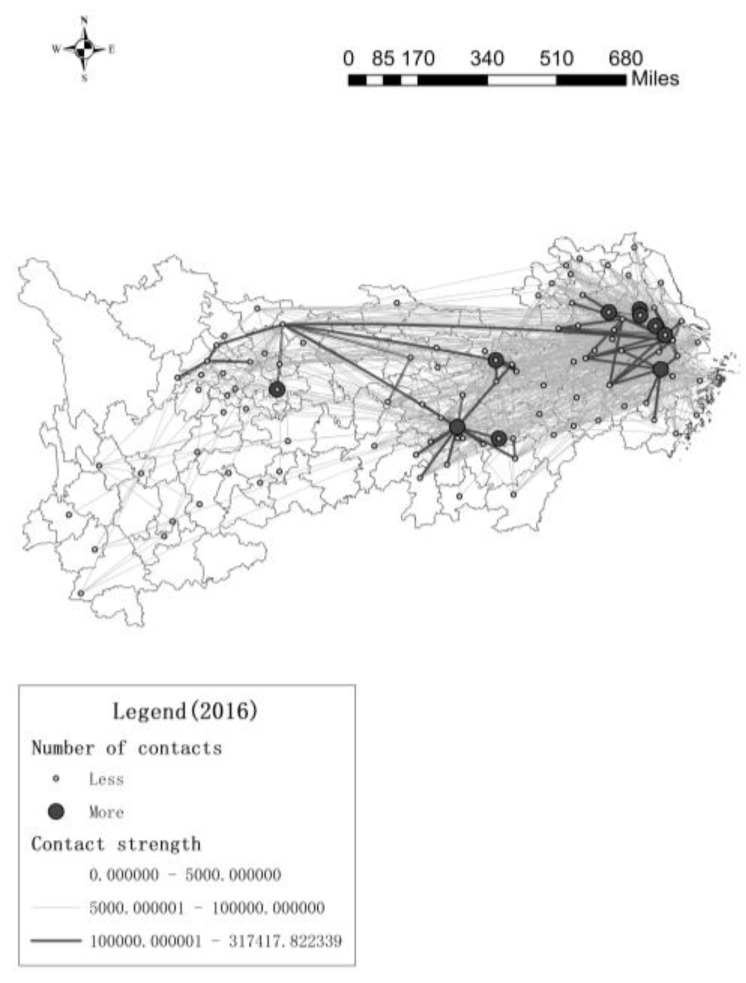
Number of urban innovation contacts in 2016.

Figure 3 and Figure 4 (They are made by “ArcGIS-xy to line”) show that the innovation contacts between the 106 cities in the Yangtze River Economic Belt, and the thick line represents more contacts, this article hides the line between the cities with lower innovation contacts.

In 2007, in the downstream area, only three cities have more innovation contacts with other cities, but they have more contacts with neighboring cities and less contact with distant cities, while there are very few innovation contacts between the midstream and upstream cities. In 2016, The innovation contact network of downstream cities continues to expand, and the number of innovation contacts continues to increase. The network with Yangzhou, Zhenjiang, Wuxi, Changzhou, Suzhou and Hangzhou as the core cities is very dense, the radiation effect and driving ability of the core cities are continuously improving, this causes the number of core cities to increase. The core cities in the midstream regional network are mainly Wuhan, Changsha and Yichun, these cities have higher centrality in the innovation network. There are few core cities in the upstream city network, but compared to 2007, the number of innovation contacts in the upstream cities has increased significantly. In addition, downstream cities not only create innovation contacts with surrounding cities, but also have more innovation contacts with cities in other river basins, such as Chengdu and Bazhong. From Figure 1 to Figure 4, maybe the more innovation contacts with other cities, the higher green innovation efficiency of cities. Next, we try to use spatial econometric model to prove this conclusion.

### 3.1. Benchmark Regression

Based on the above model setting and testing ideas, Stata software is used to construct the optimal econometric model to explore the correlation between the city centrality and green innovation efficiency, the regression results are presented in Table 2. The results of the spatial autocorrelation test indicate that the spatial econometric model is suitable for this study.

The OLS regression constitutes only a “flat domain” estimation of the parameters, which fails to reflect the spatial instability of the parameters in different spaces. Thus, the three spatial econometric models incorporating spatial correlation are selected for analysis. The Hausman test shows that all three models use fixed effects, in terms of the model-fitting effect, the SDM (Spatial Dubin Model) has higher R-sq than the SLM (Spatial Lag Model) and SEM (Spatial Error Model), while AIC (Akaike Information Criterion) and BIC (Bayesian Information Criterion) are significantly lower. To further judge the fitting effect of the SDM model, this paper performs the LR test on the SDM model, the *p* values of the corresponding LR space lag test and the LR spatial error test are significant at the 1% level, thus the SDM model cannot be converted into the SLM model or the SEM model. Based on this finding, this paper selects the spatial SDM model for analysis. Table 2 reports the regression results of SDM model (the regression results of the SLM model and the SEM model are shown in Appendix A). Control variables are not added to Model 1, and Model 2 is the fixed effect model with control variables.

In model (1), the regression coefficient estimate of DC is significantly positive, city centrality has a positive role in promoting green innovation efficiency, but the regression coefficient estimate of $DC^{2}$ is negative, which indicates that there is an inverted U-shaped relationship between city centrality and green innovation efficiencyIn other words, when the city centrality is too large, it will lead to a decline in green innovation efficiency. In addition, we define the impact of city centrality on the green innovation efficiencies of other cities as the neighboring effect of city centrality—the regression coefficient estimate of W·DC is significantly positive, and the regression coefficient estimate of $W\cdot DC^{2}$ is significantly negative, after adding other control variables that affect the green innovation efficiency, the inverted U-shaped effect is more obvious. To sum up, moderate city centrality helps improve the green innovation efficiency in cities and surrounding cities, but when city centrality is too large, this effect is “harmful to others without benefiting oneself”, that is, it will inhibit their own and surrounding cities’ green innovation efficiency. The reason for this conclusion may be that the cities with lower centrality are less connected with other cities, the spillover of innovation is limited, which is not conducive to knowledge spillover or resource acquisition and hinders the development of technological innovation activities. Cities with higher centrality are more active in innovation activities with other cities, which reduces the cost of knowledge search, and it is more conducive to the acquisition of heterogeneous resources and the development of technological innovation activities. More importantly, the core cities in the innovation network can use their “prestige” and “status” to be learned and followed by neighboring cities, which is more conducive to the accumulation of knowledge and the improvement of overall innovation efficiency. However, the heterogeneity of innovation performance comes from the ability to acquire and absorb the elements of innovation input, when the centrality of the city is too high, it is difficult for cities with high centrality to fully absorb and utilize too much innovation elements, in addition, there would be siphon effects on the surrounding cities, which limits the green innovation efficiency of the surrounding cities.

For example, many years ago, after the strategy for the rise of the central region was proposed, the overall innovation network of central cities was initially formed. Wuhan, Chengdu, Nanchang, and their surrounding cities developed rapidly, an urban circle with large cities driving small cities have been formed. But now, Wuhan is in an absolutely central position in the central region innovation network, howeve, its radiative effect on the surrounding areas is limited. The development of areas such as Huangshi, Xiaogan, and Xianning lags far behind Wuhan. These cities have not been affected by Wuhan’s knowledge spillover, instead, more talents returned to Wuhan to find work. In addition, Wuhan has the largest number of college students in China, but students from top universities are often reluctant to stay in this city after graduation or go to nearby cities. Therefore, Wuhan cannot absorb these innovation elements, which directly inhibits Wuhan’s innovation level.

### 3.2. Regional Heterogeneity

This article examines the different effects of city centrality on heterogeneous regions (upstream cities, midstream cities and upstream cities). Table 3 reports the regression results. Model (1), model (3) and model (5) respectively represent the regression without control variables, while the remaining three models contain control variables, in which the regression coefficient estimate of DC in each model are positive. Both are significant at the 1% level, but their influence on the upstream, midstream and downstream regions decreases gradually. This shows that the green innovation efficiency in the upstream region is more sensitive to the city centrality. In addition, in the upstream cities, the regression coefficient estimate of $DC^{2}$ is approximately equal to 0, which indicates that the centrality of the upstream city is positively correlated with the green innovation efficiency. The industries in the upstream cities are mostly labor-intensive industries, these industries mainly require low-skilled labor, and there are fewer jobs for high-tech talents, so there is less innovation interaction and knowledge exchange in upstream areas, there is much room for improvement in centrality and green innovation efficiency. The regression coefficient estimate of W·DC is the largest in the midstream cities, this may be due to the midstream cities, as an important base to undertake industrial transfer in the downstream cities, has a strong ability to learn and absorb innovation, it is faster to improve their green innovation efficiency with the help of the spillover effect of the downstream cities.

### 3.3. Mediation Effect Test

According to the three steps of the mediation effect test, Table 4 reports this result. Both the regression coefficient estimate of the core explanatory variable (DC) in the model (1) and model (2) are significantly positive, so city centrality positively influence the number of migrants; in model (3), the regression coefficient estimates of the core explanatory variables (DC) and mediator variable (PFL) are positively significant, and the former is smaller than the latter, which fully affirms the partially mediation effect of PFL, this result is similar to China’s actual national conditions. Due to the disappearance of China’s “demographic dividend” and the sharp decline in the number of labor forces, major cities, especially first-tier and second-tier cities, have lowered their settlement threshold, which directly affects the concentration of labor. High-level talents have flowed into more innovative cities based on the principle of “voting with their feet,” and these cities are often more central in the innovation network. The concentration of talents has further improved the city’s innovation efficiency. In addition, the regression coefficient estimate of W·PFL is significantly negative, which indicates that a large number of talents have flowed into the central city, the surrounding cities may be difficult to innovate because of the lack of necessary talents, so the flow of migrants has limited the green innovation efficiency of the surrounding cities.

The cities with higher centrality enhance the green innovation efficiency by attracting the migrants that come from cities with lower centrality, but the negative effect is that the inflow of migrants into the central city limits the green innovation efficiency of surrounding cities. We think that the possible reason is that talents are the first element of innovation, cities with more high-level talents having the first mover advantage of innovation and development. In China, cities attract high-level talents through various policies, such as providing free housing for excellent talents, providing research funding for young scholars with excellent research ability, and rewarding enterprises and innovation teams that introduce high-level talents, these policies provide core guarantee for talents. In the cities with high city centrality, the supporting policies for talents are more attractive, so more talents choose to enter these cities, which directly promotes the green innovation efficiency.

## 4. Discussion

In summary, both hypothesis 1 and hypothesis 2 are true. This paper uses social network analysis and spatial econometric models to explore the impact of city centrality on the green innovation efficiency, and gravity model is used to prove that the number of migrants is a mediator variable in the effect of city centrality promoting green innovation efficiency. The conclusions of this article will contribute to expanding existing research in two aspects. On the one hand, although previous scholars have carried out in-depth research on city innovation networks, these studies mainly explored the evolution trend of city innovation networks and the influencing factors of city innovation networks [41,42,43]. No scholars have analyzed whether the structure of city innovation networks will affect innovation. In addition, existing research generally believes that higher degree centrality reduces the cost of acquiring knowledge and talent for network members, so the degree centrality positively affects innovation performance [25]. However, this article concludes that there is an inverted U-shaped relationship between city centrality and green innovation efficiency, which makes up for the lack of analysis of the positive and negative effects of centrality in the existing literature. On the other hand, this paper uses the modified gravity model to calculate the number of migrants, cities with high city centrality will attract a large number of migrants that come from cities with low city centrality to improve the green innovation efficiency. Previous research believes that communication and cooperation between enterprises can be used to create dynamic capabilities [44], which bring higher level of innovation and competitive advantage. Enterprises in the center of knowledge network have more ways to obtain innovation resources [45]. When an enterprise uses external resources for innovation, the ability to absorb and use these resources will affect its innovation performance [46]. And the closer an enterprise is to the center of the knowledge network, the more resources it can absorb and use [47]. This article uses data from 106 cities to prove that this conclusion also apply to city innovation networks. People are important elements of innovation, when city centrality is low, there is less innovation interaction between cities, so there is no innovation spillover, the green innovation efficiency of cities is low. When city centrality is high, cities with high city centrality absorb migrants to improve the green innovation efficiency. Although the number of migrants of cities with low city centrality reduced, there is innovation spillover effect between cities, this spillover effect will bring external economy to the cities with low centrality, which is not generated inside the cities, but comes from the cities with high centrality, the innovation interaction between cities is beneficial to the increase of overall green innovation efficiency. However, when city centrality is too high, the cities with high city centrality absorb too many migrants from the cities with low centrality, but it is difficult to absorb and make good use of these innovative elements, which limits the green innovation efficiency, although there is still innovation spillover effect between cities, the cities with low centrality are difficult to innovate due to the lack of innovative elements, so their green innovation efficiency dropped. It should be noted that the endogeneity between innovation network structure and innovation is one of the limitations of this article. We hope to consider the impact of innovation network structure on innovation with the method of causal inference that weakens the endogenous problem. This represents the direction of our future work, which includes not only networks between cities, but also network interactions between and within city groups.

To check the robustness of the empirical results, the spatial adjacency weight matrix is used to replace the spatial distance weight matrix to test the benchmark model and mediation effect test [48], this helps to more carefully observe the degree of influence of the independent variable on the dependent variable and its spatial spillover effect. WL is the spatial adjacency weight matrix (If city *i* and city *j* are adjacent, each element in the matrix is equal to 1, otherwise it is 0).

The results are shown in Table 5. Model (1) and model (2) report the robustness test results of benchmark regression, models (3)–(5) report the robustness test results of mediation effect test. The direction and significance of the regression coefficient estimates of each explanatory variable are substantially consistent with the results of the benchmark regression and mediation effect test. Thus, the results of the model are robust.

There are many cases in China to prove these conclusion, for example, in the Wuhan city circle of China, the innovation level, GDP and employment of Wuhan are at the forefront. However, the development of the surrounding areas such as Xiaogan, Xianning and Xiantao is limited, and the gap with Wuhan is very obvious. The reason is that most of the high-tech migrants or low-skilled migrants in the cities around Wuhan have entered Wuhan to find jobs, which has greatly limited the innovation of surrounding cities. In addition, in the Beijing-Tianjin-Hebei city circle, Beijing’s population inflow is very high, but this has not increased the population inflow of surrounding provinces and cities (Tianjin and Hebei), and it is difficult for Beijing’s basic public facilities to provide supporting public services for such a large immigrant population, that is, it is difficult for Beijing to absorb these innovation factors, so the Xiongan New District with a non-capital evacuation function was established to undertake the population transfer in Beijing.

## 5. Conclusions

This paper draws the following conclusions. First, from 2007 to 2016, there are more and more innovation contacts among 106 cities in the Yangtze River Economic Belt, and the innovation network is becoming more and more dense. The core cities of the downstream innovation network are mainly Yangzhou, Zhenjiang, Wuxi, Changzhou, Suzhou and Hangzhou; the core cities in the midstream are mainly Wuhan, Changsha and Yichun; the core cities in the upstream are Chengdu and Bazhong.

Second, there is an inverted U-shaped relationship between city centrality and green innovation efficiency. In addition, the influence curve of centrality on the green innovation efficiency of surrounding cities also presents inverted U-shaped. Cities with high city centrality will attract a large number of migrants that come from cities with low city centrality to improve the green innovation efficiency, but the green innovation efficiency of cities with low city centrality will decline due to lack of talents. Therefore, proper city centrality can promote the green innovation efficiency to the greatest extent.

Based on the above conclusions, this paper proposes the following policy recommendations. First, we should pay attention to urban planning and construction from the perspective of network. Regional development depends more on the division of labor and cooperation between cities, the status of cities in the whole system depends not only on their own attribute characteristics, but also on their relations with other cities. Therefore, when planning regional urban development, The government should examine the status and function of the city in the whole urban system from the perspective of urban network, pay attention to the external connection of the city, reasonably determine the orientation of urban development, avoid resource waste and repeated construction in the region; vigorously develop the urban economic zone, constantly support, cultivate and improve the network of urban economic zone based on the central city, and form a certain urban network structure through the aggregation and diffusion of the central city.

Second, innovation connections have become the key of local or urban development. To achieve this kind of connection, the government not only needs to establish the local network within the region, but also needs to integrate the local economy into the global market and connect the external non local network. By using new information and network technology to improve its connection with other cities and enhance its accessibility, city centrality and technological innovation in the city innovation network can be greatly improved.

Finally, we should strengthen the existing urban center position, cultivate new innovation growth poles and promote the diffusion of knowledge and human capital, but we must avoid the waste of human capital caused by the extremely centrality of the city. Each city must not lower the threshold to settle down without a limit, because this will cause “crowding of talents” and is not conducive to the improvement of urban green innovation efficiency.

## Figures and Tables

**Table 1:** Descriptive statistics for the data.

Variable	Obs	Mean	Std. Dev.	Min	Max
id	1060	53.500	30.613	1.000	106.000
year	1060	2011.500	2.874	2007.000	2016.000
ER	1060	84.243	19.216	0.490	143.240
IS	1060	36.957	7.972	20.660	69.780
PCGDP	1060	37,418.470	27,356.270	99.000	199,017.000
GOV	1060	3,115,894.000	5,230,195.000	178,440.000	69,200,000.000
GROUP	1060	1.943	0.725	1.000	3.000
X1	1060	13,100,000.000	15,800,000.000	633,233.000	1.720 × 10^8^
X2	1060	57.207	90.059	6.290	951.850
X3	1060	82,373.190	247,911.400	894.000	3,417,109.000
X4	1060	816,924.800	1,572,814.000	12,397.000	14,900,000.000
Y1	1060	4495.692	9853.020	2.000	90,771.000
Y2	1060	2341.406	7302.548	0.118	211,450.000
y1	1060	25,731.360	55,951.860	849.000	1,347,367.000
y2	1060	8591.285	10,953.710	88.000	80,468.000
y3	1060	53,060.710	63,891.630	1709.000	682,922.000

**Table 2:** Benchmark regression.

	(1)	(2)			DC
	GIE	GIE	Geary’ C	2007	1.364 ***
DC	0.949 ***	1.227 ***			(0.000)
	(0.056)	(0.054)		2008	1.330 ***
DC^2^	−0.016 ***	−0.026 ***			(0.000)
	(0.003)	(0.002)		2009	1.288 ***
ER		0.018			(0.000)
		(0.036)		2010	1.120 ***
PCGDP		0.029			(0.000)
		(0.050)		2011	1.232 ***
GOV		−0.253 ***			(0.000)
		(0.062)		2012	1.211 ***
IS		0.761 ***			(0.000)
		(0.147)		2013	1.365 ***
Spatial rho	0.618 ***	−0.434 ***			(0.000)
	(0.053)	(0.116)		2014	1.362 ***
sigma2_e	0.095 ***	0.074 ***			(0.000)
	(0.004)	(0.003)		2015	1.219 ***
Wx					(0.000)
DC	0.355 **	1.793 ***		2016	1.227 ***
	(0.173)	(0.248)			(0.000)
DC^2^	−0.037 ***	−0.077 ***			
	(0.007)	(0.009)			
ER		2.698 ***			
		(0.628)			
PCGDP		1.611 ***			
		(0.196)			
GOV		−1.702 ***			
		(0.173)			
IS		0.931 **			
		(0.289)			
N	1060	1060			
R-sq	0.774	0.840			
AIC	528.2	275.4			
BIC	558.0	344.9			
Controlled	no	yes			

Note: *, ** and *** denote significance at the 10%, 5%, and 1% levels, respectively, and Z values are in parentheses.

**Table 3:** Heterogeneity test.

	(1)	(2)	(3)	(4)	(5)	(6)
	GIE	GIE	GIE	GIE	GIE	GIE
Main	Upstream cities		Midstream cities		Downstream cities	
DC	1.104 ***	1.100 ***	0.638 ***	0.994 ***	0.972 ***	0.940 ***
	(0.073)	(0.074)	(0.074)	(0.067)	(0.168)	(0.170)
DC^2^	−0.010 **	−0.009 **	−0.004	−0.015 ***	−0.012	−0.010
	(0.004)	(0.004)	(0.003)	(0.003)	(0.007)	(0.008)
ER		−0.052		−0.033		−0.084
		(0.072)		(0.030)		(0.438)
PCGDP		0.161 **		−0.116		−0.026
		(0.053)		(0.106)		(0.155)
GOV		−0.057		0.025		−0.130
		(0.087)		(0.111)		(0.111)
IS		0.460 *		0.335 **		0.026
		(0.248)		(0.169)		(0.590)
Wx						
DC	−0.983 ***	−0.080	0.361 *	1.936 ***	0.750 *	1.129 **
	(0.260)	(0.364)	(0.185)	(0.259)	(0.436)	(0.491)
DC^2^	0.007	−0.026 *	−0.036 ***	−0.071 ***	−0.048 **	−0.063 ***
	(0.012)	(0.016)	(0.008)	(0.010)	(0.017)	(0.019)
ER		1.197 **		−1.081 **		0.969
		(0.377)		(0.462)		(1.414)
PCGDP		0.293 *		3.518 ***		0.529
		(0.161)		(0.394)		(0.646)
GOV		−0.788 ***		−3.466 ***		−0.508
		(0.194)		(0.336)		(0.511)
IS		−0.152		0.565 **		2.591 **
		(0.380)		(0.243)		(1.198)
Spatial rho	0.811 ***	0.549 ***	0.703 ***	−0.876 ***	0.245	0.019
	(0.049)	(0.113)	(0.050)	(0.134)	(0.156)	(0.196)
sigma2_e	0.063 ***	0.058 ***	0.054 ***	0.037 ***	0.073 ***	0.070 ***
	(0.005)	(0.005)	(0.003)	(0.002)	(0.007)	(0.006)
N	310	310	500	500	250	250
R-sq	0.856	0.903	0.801	0.902	0.829	0.843
AIC	52.27	29.31	−17.54	−198.5	67.54	72.58
BIC	74.69	81.62	7.748	−139.5	88.67	121.9
Controlled	no	yes	no	yes	no	yes

Note: *, ** and *** denote significance at the 10%, 5%, and 1% levels, respectively, and Z values are in parentheses.

**Table 4:** Mediation effect test.

	(1)	(2)	(3)
	GIE	PFL	GIE
DC	1.227 ***	0.246 ***	1.178 ***
	(0.054)	(0.015)	(0.054)
DC^2^	−0.026 ***		−0.022 ***
	(0.002)		(0.002)
PFL			2.332 ***
			(0.429)
_cons		−5.989 ***	
		(0.169)	
Wx			
DC	1.793 ***		0.204
	(0.248)		(0.312)
DC^2^	−0.077 ***		−0.009
	(0.009)		(0.013)
PFL			−10.512 ***
			(1.424)
ER	2.698 ***		1.360 **
	(0.628)		(0.641)
PCGDP	1.611 ***		2.629 ***
	(0.196)		(0.282)
GOV	−1.702 ***		−1.241 ***
	(0.173)		(0.175)
IS	0.931 **		0.644 **
	(0.289)		(0.281)
rho	−0.434 ***		0.033
	(0.116)		(0.122)
sigma2_e	0.074 ***		0.069 ***
	(0.003)		(0.003)
N	1060	1060	1060
R−sq	0.840	0.229	0.854
AIC	275.4	2534.9	209.5
BIC	344.9	2544.8	288.9

Note: *, ** and *** denote significance at the 10%, 5%, and 1% levels, respectively, and Z values are in parentheses.

**Table 5:** Results of the robustness test.

	(1)	(2)	(3)	(4)	(5)
	GIE	GIE	GIE	PFL	GIE
Main	WL	WL	WL		WL
DC	1.043 ***	1.076 ***	1.076 ***	0.246 ***	1.126 ***
	(0.056)	(0.052)	(0.052)	(0.015)	(0.051)
DC^2^	−0.018 ***	−0.019 ***	−0.019 ***		−0.021 ***
	(0.003)	(0.002)	(0.002)		(0.002)
PFL					3.329 ***
					(0.438)
_cons				−5.989 ***	
				(0.169)	
Wx					
DC	−0.195 **	0.206 **	0.206 **		0.175 *
	(0.095)	(0.096)	(0.096)		(0.096)
DC^2^	−0.010 **	−0.018 ***	−0.018 ***		−0.018 ***
	(0.004)	(0.004)	(0.004)		(0.004)
PFL					−2.210 ***
					(0.614)
ER		0.098	0.098		0.107
		(0.083)	(0.083)		(0.081)
PCGDP		0.152	0.152		0.086
		(0.099)	(0.099)		(0.104)
GOV		−0.381 ***	−0.381 ***		−0.380 ***
		(0.086)	(0.086)		(0.090)
IS		−0.028	−0.028		−0.150
		(0.190)	(0.190)		(0.190)
Spatial rho	0.379 ***	0.083 **	0.083 **		0.118 **
	(0.032)	(0.040)	(0.040)		(0.040)
sigma2_e	0.097 ***	0.082 ***	0.082 ***		0.078 ***
	(0.004)	(0.004)	(0.004)		(0.003)
N	1060	1060	1060	1060	1060
R−sq	0.763	0.826	0.826	0.282	0.834
AIC	591.7	391.9	391.9	2345.7	339.8
BIC	621.5	461.4	461.4	2355.6	419.2
Controlled	no	yes	yes	no	yes

Note: *, ** and *** denote significance at the 10%, 5%, and 1% levels, respectively, and Z values are in parentheses.

**Figure A1:**
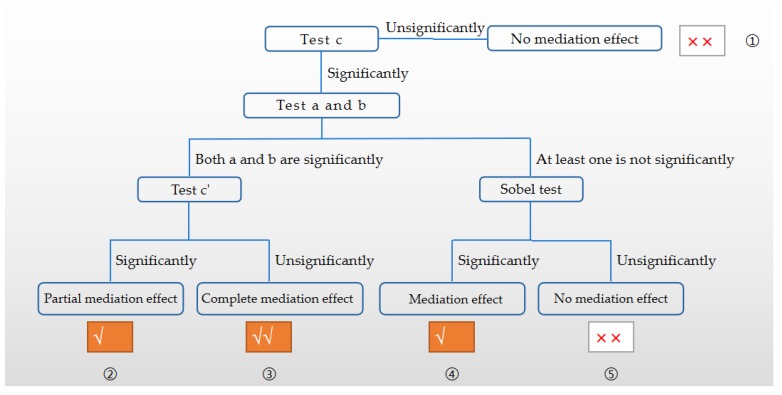
Judgment conditions for mediation effect test.

## Appendix A

### Appendix A.1. Spatial Autocorrelation Test

Geary’ C:

$$C=\frac{(n-1)\sum_{i=1}^{n}\sum_{j=1}^{n}W{\left(x_{i}-x_{j}\right)}^{2}}{2\sum_{i=1}^{n}\sum_{j=1}^{n}W\sum_{i=1}^{n}{\left(x_{i}-x_{j}\right)}^{2}}$$

n represents the number of cities, W represents spatial weight matrix, x represents variable what we want to test, C represents Geary’s C. When the regression coefficient estimate of Geary’s C is significant, indicating that there is spatial autocorrelation, we can use a spatial econometric model.

**Table A1:** Spatial autocorrelation test.

		DC	GIE	PFL
Geary’ C	2007	1.364 ***	1.099 ***	1.214 ***
		(0.000)	(0.002)	(0.000)
	2008	1.330 ***	1.075 ***	1.214 ***
		(0.000)	(0.015)	(0.000)
	2009	1.288 ***	1.245 ***	1.211 ***
		(0.000)	(0.001)	(0.000)
	2010	1.120 ***	1.194 ***	1.213 ***
		(0.000)	(0.001)	(0.000)
	2011	1.232 ***	1.092 **	1.217 ***
		(0.000)	(0.029)	(0.000)
	2012	1.211 ***	1.077 **	1.214 ***
		(0.000)	(0.019)	(0.000)
	2013	1.365 ***	1.077 ***	1.217 ***
		(0.000)	(0.004)	(0.000)
	2014	1.362 ***	1.405 ***	1.214 ***
		(0.000)	(0.005)	(0.000)
	2015	1.219 ***	1.058 *	1.214 ***
		(0.000)	(0.253)	(0.000)
	2016	1.227 ***	1.023	1.211 ***
		(0.000)	(0.344)	(0.000)

Note: *, ** and *** denote significance at the 10%, 5%, and 1% levels, respectively, and Z values are in parentheses.

### Appendix A.2. Benchmark Regression

**Table A2:** Benchmark regression.

	SLM		SEM	
	(1)	(2)	(3)	(4)
	lnGIE	lnGIE	lnGIE	lnGIE
DC	1.064 ***	1.098 ***	0.812 ***	0.915 ***
	(0.067)	(0.055)	(0.053)	(0.054)
DC^2^	−0.029 ***	−0.023 ***	−0.010 ***	−0.014 ***
	(0.003)	(0.002)	(0.002)	(0.003)
ER		−0.032		−0.044
		(0.040)		(0.039)
PCGDP		−0.118 **		−0.199 ***
		(0.048)		(0.050)
GOV		−0.498 ***		−0.434 ***
		(0.039)		(0.050)
IS		0.732 ***		0.854 ***
		(0.127)		(0.153)
Spatial rho	0.011	−0.093 **		0.852 ***
	(0.043)	(0.037)		(0.159)
lambda			0.870 ***	0.580 ***
			(0.024)	(0.087)
sigma2_e	0.137 ***	0.093 ***	0.098 ***	0.090 ***
	(0.006)	(0.004)	(0.004)	(0.004)
N	1060	1060	1060	1060
R−sq	0.710	0.807	0.696	0.799
AIC	912.9	502.6	580.8	473.2
BIC	932.8	542.3	600.6	513.0
Controlled	no	yes	no	yes

Note: *, ** and *** denote significance at the 10%, 5%, and 1% levels, respectively, and Z values are in parentheses.

### Appendix A.3. Explanation of the Mediation Effect

To help reviewers better understand the mediation effect test, we have detailed the testing process of the mediation effect.

(A1) $$Y=cX+e_{1}$$

(A2) $$M=aX+e_{2}$$

(A3) $$Y=c^{\prime }X+bM+e_{3}$$

where the coefficient (c) of Equation (A1) is the total effect of independent variable (X) on dependent variable (Y); the coefficient (a) of Equation (A2) is the effect of independent variable (X) on mediator variable (M); coefficient (b) of Equation (A3) is the effect of mediator variable (M) on dependent variable (Y) after controlling the influence of independent variable (X); the coefficient ($c^{\prime }$) is the direct effect of the independent variable on the dependent variable (Y) after controlling the effect of the mediator variable (M); $e_{1}\sim e_{3}$ are the regression residual. For such a simple mediation model, the mediation effect is equal to the indirect effect, that is, equal to the coefficient product (ab), and it has the following relationship with the total effect and the direct effect (MacKinnon, Warsi, & Dwyer, 1995):

(A4) $$c=c^{\prime }+ab$$

The judgment conditions of mediation effect are shown in Figure A1.

The most commonly used method to test the mediation effect is to test the regression coefficients step by step (Baron & Kenny, 1986; Judd & Kenny, 1981). First, test the coefficient (c) of Equation (A1). Second, test the coefficient (a) of Equation (A2) and the coefficient (b) of Equation (A3) in turn. If both a and b are significant, than we test $c^{\prime }$, if the regression coefficient estimate of $c^{\prime }$ is significantly, M has a partial mediation effect. If the regression coefficient estimate of $c^{\prime }$ is not significantly, then M has a complete mediation effect. If at least one of a and b is not significant, then we should do Sobel test.

In our article, the result is the same as the second case in the Figure A1, Y is equivalent to GIE, X is equivalent to DC, M is equivalent to PFL. Furthermore, in Table 4 in our article, the regression coefficient estimate of the core explanatory variables (DC) is smaller than the regression coefficient estimate of mediator variable (PFL), which fully affirms the partially mediation effect of PFL.
